# The contribution of the two hemispheres to lexical decision in different languages

**DOI:** 10.1186/1744-9081-8-3

**Published:** 2012-01-09

**Authors:** Raphiq Ibrahim, Zohar Eviatar

**Affiliations:** 1 The Edmond J. Safra Brain Research Center for the Study of Learning Disabilities and Learning Disabilities Department, University of Haifa, Haifa, Israel; 2 Institute for Information Processing and Decision Making (IIPDM) and Psychology Department, University of Haifa, Haifa, Israel

## Abstract

**Background:**

Both reading words and text in Arabic is slower than in other languages, even among skilled native Arabic speakers Previously we have shown that the right hemisphere (RH) had difficulty in matching Arabic letters, and suggested that it cannot contribute to word recognition in Arabic. In this study we tested this finding directly.

**Method:**

We used the Divided Visual Field (DVF) lexical decision (LD) paradigm to assess hemispheric function during reading. The experiment had two conditions (unilateral and bilateral). In the unilateral condition, the target stimulus was presented unilaterally to the left or the right visual field. In the bilateral condition two stimuli were presented simultaneously, and participants were cued as to which one was the target. Three groups of participants were tested: Arabic speakers, Hebrew speakers, and English speakers. Each group was tested in their native language.

**Results:**

For Hebrew and English speakers, performance in both visual fields was significantly better in the unilateral than in the bilateral condition. For Arabic speakers, performance in the right visual field (RVF, where stimuli are presented directly to the left hemisphere) did not change in the two conditions. Performance in the LVF (when stimuli are presented directly to the right hemisphere) was at chance level in the bilateral condition, but not in the unilateral condition.

**Conclusion:**

We interpret these data as supporting the hypothesis that in English and Hebrew, both hemispheres are involved in LD, whereas in Arabic, the right hemisphere is not involved in word recognition.

## Background

An examination of the differences in orthography/phonology relations among languages and participants' language experience, reveals that the processing of Arabic orthography seems to make different demands on the cognitive system of beginners [1] as well as skilled readers [2]. In previous research, we have suggested that this happens because Arabic orthography specifically disallows the involvement of the right hemisphere (RH) in letter identification, even while the RH of the same participants contributes to this process in English and Hebrew [3,4].

This hypothesis may also be supported by the recent finding of Abdulhadi, Ibrahim and Eviatar, that children who are considered good readers by their teachers in 6^th ^grade, do not show a word superiority effect in a vowel detection task in Arabic [5]. This is the consistent finding that among literate participants, letters are detected faster in the context of real words than in psuedo-words [6]. The usual explanation for this effect is that real words are recognized quickly via their global features, and their constituents (the letters) can be inferred quickly, whereas nonwords, being novel stimuli, require sequential letter-by-letter processing. The fact that the 6^th ^graders failed to detect a vowel diacritic faster in the context of a real word than in the context of a nonsense word suggested to us that they do not employ a global word-form strategy. If words, nonwords, and nonletter stimuli are processed similarly, this may indicate a low level of automatization of the reading process. We hypothesize that this too may be a result of lower levels of RH particpation in normal reading than in other languages. This hypothesis is related to a recent debate in the literature over the degree of interhemispheric interaction that is necessary during normal reading of fixated words [6,7]. If it is the case that normal reading always requires interhemispheric integration of information, and if it is the case that the right hemisphere is deficient in processing orthographic information in Arabic, this may explain the difficulties in reading. In the following, we first review the characteristics of Arabic orthography, and then present the logic of our design, which allowed us to examine dependent and independent hemispheric functions.

## Arabic language and orthography

Arabic is a Semitic language, written in a consonantal alphabet. Arabic orthography is read and written from right to left. Arabic has a rich morphology [8] which is based largely on a concatenate "root-and-pattern" [9]. The roots generally consist of three or four consonants and give the basic lexical meaning of the word [10,11]. The pattern (noun-form or verb-form) bring specific grammatical information such as number, tense, person, gender etc. Written Arabic has two versions: a *shallow orthography*, in which short vowels can be indicated by using diacritical marks, such as dots and dashes that appear below, above or inside the consonantal base of the word; and a *deep orthography *where the diacritics indicating short vowels are omitted [12,7].

Shallow orthographies have the advantage of ensuring efficient acquisition of the reading and writing process [13]. Share (2008) has termed this feature "decipherability" [14]. In Semitic orthographies, vowel signs of all kinds provide phonological information and allow a simple process of grapheme-to-phoneme conversion, which potentially facilitates word recognition by specifying the correct pronunciation of the written word [13,15]. For instance, in Arabic pointed orthography, there are unambiguous grapheme-to-phoneme relations: كَتَبَ - kataba (wrote) has one reading option, while the unpointed orthography in which the grapheme-phoneme relation is ambiguous, the unpointed word كتب (ktb) has a number of reading options: كُتِبَ - kotiba (had been written); كَتَبَ - kataba (wrote); كُتُب - kotob (books). It is important to note that the diacritical marks not only convey phonological cues that help disambiguate homographs and provide word meaning [16], but they also have grammatical functions [7], helping the reader determine whether the word is a verb كَتَبَ - kataba (wrote) or a noun كُتُب - kotob (books).

Additionally, there are unique orthographic and linguistic characteristics which may make the task of reading Arabic even more complex. First, 23 of the 29 letters in the alphabet have four shapes each (word initial, medial, final, and when they follow a non-connecting letter, for example, the phoneme/h/is represented by the graphemes: , and six letters have two shapes each, final and separate, such that the same phoneme is represented by different graphemes, and similar graphemes representing quite different phonemes (for example, the graphemes:  represent the phonemes/t/,/b/,/y/, and /n/, respectively). Second, the majority of letters are connected to their neighbors from both sides (right and left), except for six letters , that are connected only from the right side. Thus, most words in the language are comprised of completely connected letters, or contain at least some connected letters, with letter strings composed of separate letters being very infrequent.

## The Logic of the Paradigm

The consensus in the field is that both of the cerebral hemispheres are involved in the process of reading [17]. The relative contribution of each hemisphere to the process constitutes a function of individual differences [18] related to the characteristics of the way in which the language is read [19,20] and other factors. One way to assess hemispheric function is using the Divided Visual Field (DVF) paradigm. In this experimental paradigm, we take advantage of the way in which the eyes are hooked up to the primary visual cortex. Stimuli that presented to the right of visual fixation are available only to the left hemisphere (LH) at the first stages of processing. Stimuli that are presented to the left of visual fixation are initially available only to the right hemisphere (RH). This contra-lateral organization has been verified by electrophysiological and imaging data [21]. Lateralized presentation of linguistic stimuli usually results in performance asymmetries where participants respond faster and more accurately to stimuli presented in the right visual field (RVF), directly to the LH, than to stimuli presented in the left visual field (LVF), directly to the RH. This performance asymmetry is taken to reflect hemispheric functioning. Variations in the perfromance asymmetry are then interpreted as variations in hemispheric functions for different types of stimuli and for different groups of participants.

In the present study we used two variations of a lateralized lexical decision (LD) task. In the unilateral condition, one stimulus (either a word or a phonotactically legal nonword) was presented in one or the other visual field. In the bilateral condition, two stimuli were presented on each trial, with one designated as the target and the other as a distractor. Comparison of performance levels in the two conditions can estimate the degree to which interhemispheric integration is necessary to perform the task. Although the targets are initially presented to a single hemisphere in both conditions, in the bilateral condition, the other hemisphere is presented with a distractor, whereas in the unilateral condition, it is free to contribute to processing. Thus, comparison of the effects of presentation of a distractor can indicate whether interhemispheric interaction affects performance. If there is no effect on performance, this is interpreted as indicating hemispheric independence, whereas a decline in performance in the bilateral condition as compared to the unilateral condition is interpreted as indicating hemispheric interdependence. Boles (1990) has suggested that bilateral presentation disrupts communication between the hemispheres, and thus is a better estimator of independent hemispehric abilities than unilateral presentations [22]. Iacoboni and Zaidel (1996) [23] used this logic to explore hemispheric involvement in LD in English, and report that performance in both visual fields was affected by presentation condition. In contrast, Finkbeiner, Almeida, and Caramazza (2006) used this paradigm with semantic decision and letter recognition tasks, also in English [24]. They showed that presenting a distractor to the LVF had no effect on the accuracy of performance in the RVF, but that presenting a distractor to the RVF resulted in a significant rise in target misses in the LVF. They concluded that word and letter recognition are performed by a specialized processor that is located in the LH, and suggested that this is the visual word form area as defined by Cohen, Martinaud, Lemer, Lehericy, Samson and Obadia (2003) [25]. Thus, both studies suggest that processing of stimuli presented directly to the RH is more accurate when the LH is not occupied by a distractor. The studies differ in the effects of presentation mode in the RVF (when stimuli were presented directly to the LH). In Iacoboni and Zaidel's LD task, although performance in the RVF was always more accurate than in the LVF, performance was significanly better in the unilateral than in the bilateral condition [23]. This could result from two scenarios: either the RH always contributes to LD, even when the stimuli are presented directly to the specialized LH, or, in the bilateral condition, LVF distractors are automatically transferred to the LH, competing with with RVF stimuli for processing. In Finkbiener, Almeida, and Caramazza's (2006) semantic decision task, performance in the RVF was not affected by what happened in the LVF, and they concluded that the LH processes RVF stimuli independently [24].

In the present experiment we used this paradigm to examine relative hemispheric contribution and interaction in a LD task in native speakers of three languages: English, Hebrew, and Arabic. Each group was tested in their native language, with half completing the unilateral condition and half the bilateral condition. Comparison of accuracy of lexical decisions bewteen these conditions can inform us about hemispheric functions in readers of these three languages. Given that it has been shown that letter identification takes longer in Arabic than in Hebrew [3], and longer in Hebrew than in English [20], we believe that response time measures would not be informative. We therefore used d' as an index of sensitivity to lexical status, and compared relative levels of sensitivity between the language groups. Use of this signal detection measure also allowed us to assess response bias in each of the conditions.

## Method

### Participants

The participants were 120 students at Haifa University, 40 in each native language group. The native English speakers were recruited from the summer Overseas Program. All were American, and were paid for their participation. The native Hebrew and Arabic speakers were all students at Haifa University. Most of them completed the experiments for course credit, with some receiving payment instead. All were right-handed, neurologically normal, and had normal or corrected vision. The results of 3 Arabic speakers were discarded due to experimenter error, such that we analyzed the results of 18 participants in the bilateral condition and 19 in the unilateral condition.

### Ethical approval

This experimental research has been performed with the approval of ethics committee at the University of Haifa.

### Stimuli

The stimuli were comprised of 80 words and 80 legal nonwords in each language. The English list was comprised of nouns and adjectives, and the Hebrew and Arabic words were all nouns. The lists were equated on the average frequency of the words and for initial letters. All of the stimuli were either 5 or six letters long. The English stimuli were presented in Times New Roman font, the Hebrew stimuli in Guttman-Miryam font, and the Arabic stimuli in MSC Madinah S U Normal font. All were in font size 22, resulting in the longest words subtending 2.5° of visual angle.

### Procedure

The participants were tested individually. The stimuli were presented on a Silicon Graphics Workstation. On each trial the sequence of events was the following: a 1000 Hz tone sounded for 100 ms to alert the participant that the trial was beginning. Then the fixation cross was presented for 100 ms. The stimuli were presented for 180 ms horizontally, with their inner edge 2° of visual angle offset from fixation. In the unilateral condition, a single stimulus was presented either in the left or the right visual field. In the bilateral condition, 2 stimuli were presented simultaneously; one of the stimuli was designated as the target by being underlined. In both conditions, the stimuli appeared between 2° and 5° offset from fixation. The stimuli were followed by a pattern-mask that remained on screen until the participant responded or 3 sec had passed. The screen was blank for 2 sec, and the next trial began. Participant responded on the keyboard by pressing the up-arrow if the stimulus was a real word and the down-arrow if it was not.

## Results

**Figure 1:**
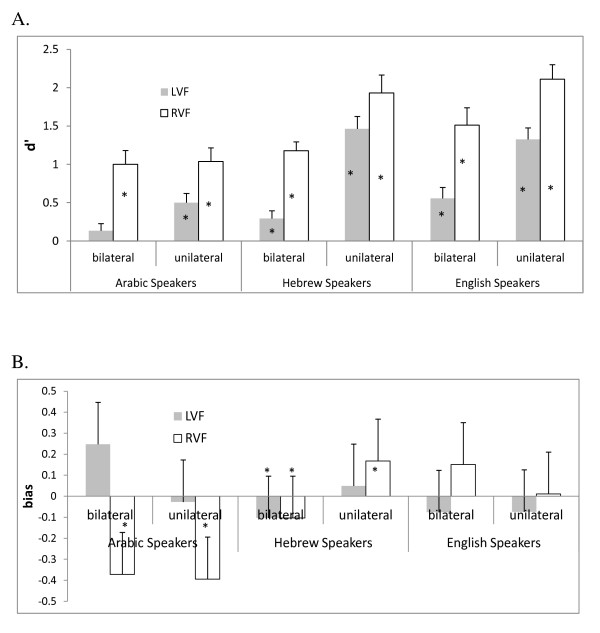
**Interhemispheric interaction for all stimuli during word recognition in Arabic, Hebrew, and English. Panel A: **Sensitivity scores (d') in the bilateral and unilateral conditions for native readers of Arabic, Hebrew, and English. * indicate that d' is significantly different from 0, such that participants were not responding at chance level. Error bars are standard errors. **Panel B**: bias scores, indexing response biases in errors. Positive number indicates misses (a bias to respond 'nonword' when the stimuli were really words) Negative scores indicate false positives (a bias to respond 'word' when the stimuli were nonwords). Error bars are standard errors.

We computed the sensitivity measure d' as the difference between the z-scores for the probability of hits (for words) and for false alarms (for nonwords), and the bias as c = (z(p(hits))+z(p(FA))/2. Thus, when bias is a negative number, this indicates a bias to respond 'yes', and where bias is a positive number, this indicates a bias to respond 'no'. We used correction computations for probability values of 1 (was changed to 1/(2N)) and 0 (was changed to 1-1/(2N)) based on the suggestions of Macmillan and Creelman (1990) [26]. These data were analyzed with native language (Arabic, English, or Hebrew) and presentation mode (unilateral or bilateral) as between groups factors, and visual field (LVF or RVF), as a within-subject factor. The cell means for both sensitivity and response bias are illustrated in Figure 1.

It can be seen that all of the particpants evinced the expected RVF advantage, reflecting LH specialization for the task. The analysis of sensitivity scores (d') revealed a significant interaction between presentation mode and visual field, F(1,111) = 4.19, η_p_^2 ^= .04, p < .05, and a significant interaction between native language and presentation mode, F(2,111) = 4.12, η_p_^2 ^= .07, p < .05. In addition, the main effects of each of the three factors were significant (visual field, F(1,111) = 101.47, η_p_^2 ^= .48, p < .001; presentation mode, F(1,111) = 32.33, η_p_^2 ^= .23, p < .001; and language, F(2,111) = 15.3, η_p_^2 ^= .22, p < .001. Planned comparisons revealed that for both Hebrew and English speakers, although the effect of presentation mode on LVF performance was very dramatic, (Hebrew speakers: F(1,39) = 40.25, η_p_^2 ^= .51, p < .0001; English speakers: F(1,39) = 14.52, η_p_^2 ^= .28, p < .001), it was also significant in the RVF (Hebrew speakers: F(1,39) = 8.70, η_p_^2 ^= .19, p < .0001; English speakers: F(1,39) = 4.3, η_p_^2 ^= .10, p < .05). For Arabic speakers, presentation mode significantly affected LVF performance, F(1,36) = 5.95, η_p_^2 ^= .15, p < .05, but not RVF performance, p > .8. One-sample t-tests were done on the d' scores to test if performance was signficantly better than chance. It can be seen in the Figure that all conditions except the LVF bilateral condition for Arabic speakers resulted in sensitivity that is greater than chance.

The analysis of response bias revealed a significant 3-way interaction between language, presentation mode, and visual field, F(2,111) = 4.15, η_p_^2 ^= .07, p < .05, a two-way interaction between language and visual field, F(2,111) = 23.95, η_p_^2 ^= .30, p < .0001; a two-way interaction between presentation mode and visual field, F(1,111) = 4.33, η_p_^2 ^= .03, p < .05; and two main effects: of visual field, F(1,111) = 12.21, η_p_^2 ^= .10, p < .0001; and language, F(2,111) = 5.62, η_p_^2 ^= .09, 12 p < .05. Planned comparisons revealed that presentation mode had no effect in either visual field for English speakers, with bias not significantly different from 0 in all conditions, except in the RVF in the bilateral condition, where the effect is marginal (p = .08). For Hebrew speakers, bias was not affected by presentation mode in the LVF, but it was in the RVF, F(1,39) = 11.83, η_p_^2 ^= .24, p < .001. It can be seen in the Figure that the bilateral presentation mode resulted in a slight and identical positive bias (to say 'yes') in both visual fields, wheres in the unilateral condition, there is a slight negative bias in both visual fields, which is significant in the RVF but not in the LVF. For Arabic speakers, we see a third pattern: response bias is not affected by presentation mode in the RVF (where it is significantly positive), but it is affected significantly in the LVF, F(1,36) = 5.52, η_p_^2 ^= .13, p < .05, where it is significantly negative in the bilateral condition.

## Discussion

The goal of the present research was to explore the relative involvement of the cerebral hemispheres in a LD task by comparing performance in each visual field when the other hemisphere is distracted (the bilateral condition) with the condition in which it is not (the unilateral condition). More specifically, we tested the hypothesis that the RH is less involved in word recognition in Arabic than in Hebrew and in English. The accuracy patterns reflected in both sensitivity and response bias support this hypothesis.

In Hebrew and English, even though there is a large performance asymmetry reflecting LH specialization for the task, performance in the RVF (LH) improves in the unilateral condition as compared to the bilateral condition. This finding replicates the results reported by Iacoboni and Zaidel (1996) [23] who also used a LD task, and diverges from the findings of Finkbeiner, Almeida, and Caramazza (2006) [24], who used a semantic decision task. In both languages, the dramatic difference in sensitivity scores occurs in the LVF, suggesting two possible scenarions, both of which include interhemispheric interaction. In the first scenario, in the unilteral condition, the RH utilizes LH resources to perform the task. In the bilateral condition these resources are not available, because the LH is processing a distractor, and thus performance levels fall. The alternative scenario posits that LVF stimuli are always transferred to the LH for processing, and that in the bilateral condition, LVF stimuli compete with the distractor, resulting in lower performance levels. However, the fact that presentation mode also affected perfromance levels in the RVF, suggests that the RH also particpates in processing of RVF stimuli. Thus, for Hebrew and English speakers, it seems that hemispheric integration occurs for all stimuli. For the Hebrew speakers, this interpretation is supported by the patterns of response bias. Response bias is different in the bilateral and unilateral conditions, but it is in the same direction in the two visual fields. For the English speakers, response bias is not informative, as all of the responses were relatively unbiased. These data support the the Split Fovea Hypothesis (see [7] for a review), that both hemispheres are always involved in reading. Our data suggest that even when stimuli are lateralized, both hemispheres are involved in LD.

The results for the Arabic speakers present a completely different pattern. The sensitivity scores reveal a large effect of presentation mode in the LVF, with performance in the bilateral condition not being different from chance, whereas performance in the unilateral condition is significantly better. Thus, in this group, it seems that without LH resources, the RH cannot perform the LD. The hypothesis that we are seeing independent RH performance in the bilateral condition is supported by the bias measure, which is negative in the LVF in this condition, and is significantly different from response bias in the unilateral condition. Most interestingly, there is no effect of presentation mode in the RVF. Thus, the LH of Arabic readers performs the LD independently of the RH, whereas the RH of Arabic readers requires LH resources in order to perfrom the task.

On the basis of these findings, we suggest that the RH is less involved in word identification in Arabic than in Hebrew and English. This hypothesis converges with other findings from our lab that explored hemispheric specialization in letter and word identification in Arabic [1,3,4]. This hypothesis suggests a neural source for the slowness of reading acquisition in Arabic as compared to other alphabetic languages.

Previously we have suggested that the characteristics of Arabic orthography are incongruent with RH abilities, such as global recognition of words. We have reported that the similarity of letters representing different phonemes, where the differences occur in very local aspects of the letters, such as the placements of dots, results in lower discrimination between them in the LVF, but not in the RVF. These findings converge with the hypothesis that the RH is more sensitive to global aspects of stimuli, while the LH is more sensitive to local aspects of stimuli [4]. In addition, we have reported that good readers in 6^th ^grade do not reveal a word superiority effect in a vowel detection task [5]. We suggested that these findings are related. The early stages of reading are characterized by serial processing of letters, the computing of their phonological value, and the combination of these parts into the whole word [25]. As children become more skilled readers, they develop a faster, parallel manner of identifying words, based on global shapes as well as on the identity of their constituent letters [27,28]. This ability is related to the development of a specific region in the fusiform gyrus at the left hemisphere, named 'the visual word form area' by McCandliss, Cohen, and Dehaene (2003) [29]. Imaging studies show that activation in this area is affected by orthography, frequency, and lexical status: the region is activated more by real words than nonsense words [30]. It may be the case that the development of this specialization in Arabic takes longer than it does in other, simpler scripts.

The vowel detection study of Abdelhadi, Ibrahim and Eviatar (2011) revealed another piece of the puzzle that can be used to examine hemispheric functioning in reading in Arabic [5]. Both 3^rd ^and 6^th ^graders showed better performance for words and nonwords that were comprised of connected letters than to words and nonwords that were comprised of unconnected letters. This probably reflects the fact that the majority of words in Arabic are made up of connected letters, so that the children had more experience with this kind of stimuli. In addition, it has been suggested that skilled readers in Arabic utilize a lexical strategy when reading, recognizing words as global patterns, rather than decoding them letter by letter [30]. It has been hypothesized that this global reading strategy is characteristic of the RH [31]. Thus, if skilled Arabic readers utilize a global word-form strategy, and if this is the strategy favored by the RH in reading, why do we find a pattern suggest that the RH in not involved in LD? It might be the case that by using lexical decision, we neutralized this strategy, and that in a stimulus set that does not include nonwords the pattern would be different. We are now in the process of testing this hypothesis.

## Conclusions

In this research, we have shown that measures of interhemispheric integration in a lexical decision task result in similar patterns among readers of Hebrew and English, despite the fact that the orthographies of these languages differ significantly, both in terms of visual complexity, morphological structure of words, and reading direction [20]. Both groups reveal patterns suggesting that both hemispheres contributed to processing of stimuli in both visual fields. The patterns shown by readers of Arabic, despite its similarity to Hebrew in terms of reading direction and morphological structure, are different, suggesting less involvement of the RH in the lexical decision task than in the other language groups.

## Abbreviations

LD: Lexical Decision; RH: Right Hemisphere; DVF: Divided Visual Field; RVF: Right Visual Field; LH: Left Hemisphere; LVF: Left Visual Field

## Competing interests

The authors declare that they have no competing interests.

## Authors' contributions

IR contributed in conception, organization, execution, writing and reviewing the manuscript. EZ contributed in conception, organization, execution and review. All authors read and approved the final manuscript.

## Consent section

Written informed consent was obtained from the participants for publication of this study.
